# Supplementary material for: Identification and functional analysis of non-coding regulatory small RNA FenSr3 in Bacillus amyloliquefaciens LPB-18
Source: PeerJ. 2023 May 15;11:e15236. doi: 10.7717/peerj.15236 (PMC10194069; doi:10.7717/peerj.15236)
Supplement: Supplemental Information 4 [file peerj-11-15236-s004.zip › KO/CK-vs-T1_map/map04964.html]

KEGG PATHWAY: Proximal tubule bicarbonate reclamation - Reference pathway


|  |  |
| --- | --- |
| **Proximal tubule bicarbonate reclamation - Reference pathway** |  |

[
Pathway menu
| Organism menu
| Pathway entry
| Show description
| User data mapping
]

One of the major tasks of the renal proximal tubule (PT) is to secrete acid into the tubule lumen, thereby reabsorbing approximately 80% of the filtered bicarbonate (HCO3(-)), as well as generating "new HCO3(-)" for regulating blood pH. In the tubular lumen, filtered HCO3(-) combines with H(+) in a reaction catalyzed by CA IV. The CO2 thus produced rapidly diffuses into the tubular cells and is combined with water to produce intracellular H(+) and HCO3(-), catalyzed by soluble cytoplasmic CA II. HCO3(-) is then cotransported with Na(+) into blood via the NBC-1. The intracellular H(+) produced by CA II is secreted into the tubular lumen predominantly via the NHE-3. The PT creates the "new HCO3(-)" by taking glutamine and metabolizing it to two molecules each of NH4(+) and HCO3(-). The NH4(+) is excreted into the tubular lumen, and the HCO3(-) , which is "new HCO3(-) ," is returned to the blood, where it replaces the HCO3(-) lost earlier in the titration of nonvolatile acids.

|  |  |  |
| --- | --- | --- |
| Reference pathway | 184% 150% 122% 100% 82% 67% 55% | 图片下载 |
